# Risk of within-hotel transmission of SARS-CoV-2 during on-arrival quarantine in Hong Kong: an epidemiological and phylogenomic investigation

**DOI:** 10.1016/j.lanwpc.2022.100678

**Published:** 2023-01-07

**Authors:** Dillon C. Adam, Mario Martín-Sánchez, Haogao Gu, Bingyi Yang, Yun Lin, Peng Wu, Eric H.Y. Lau, Gabriel M. Leung, Leo L.M. Poon, Benjamin J. Cowling

**Affiliations:** a WHO Collaborating Centre for Infectious Disease Epidemiology and Control, School of Public Health, Li Ka Shing Faculty of Medicine, The University of Hong Kong, Hong Kong Special Administrative Region, China; b Laboratory of Data Discovery for Health Limited (D^*2*^4H), Hong Kong Science and Technology Park, Hong Kong Special Administrative Region, China; c HKU-Pasteur Research Pole, School of Public Health, LKS Faculty of Medicine, The University of Hong Kong, Hong Kong, China; d Centre for Immunology & Infection, Hong Kong Science and Technology Park, Hong Kong, China

## Abstract

**Background:**

On-arrival quarantine has been one of the primary measures to prevent the introduction of SARS-CoV-2 into Hong Kong since the start of the pandemic. Most on-arrival quarantines have been done in hotels, with the duration of quarantine and testing frequency during quarantine modified over time along with other pandemic control measures. However, hotels are not designed with infection control in mind. We aimed to systematically study the potential risk of acquisition of SARS-CoV-2 infection among individuals undergoing hotel quarantine.

**Methods:**

We examined data on each laboratory-confirmed COVID-19 case identified in on-arrival quarantine in a hotel in Hong Kong between 1 May 2020 and 31 January 2022. We sequenced the whole genomes of viruses from cases that overlapped with other confirmed cases in terms of the hotel of stay, date of arrival and date of testing positive. By combining multiple sources of evidence, we identify probable and plausible transmission events and calculate the overall risk of transmission.

**Findings:**

Among 221 imported cases that overlapped with other cases detected during hotel quarantine with available sequence data, phylogenomic analyses identified five probable and two plausible clusters of within-hotel transmission. Only two of these clusters were recognised at the time. Including other clusters reported in Hong Kong, we estimate that 8–11 per 1000 cases identified in hotel quarantine may be infected by another unlinked case during quarantine, or 2–3 per 100,000 overseas arrivals.

**Interpretation:**

We have identified additional undetected occurrences of COVID-19 transmission within hotel quarantine in Hong Kong. Although hotels provide suboptimal infection control as improvised quarantine facilities, the risk of contracting infection whilst in quarantine is low. However, these unlikely events could have high consequences by allowing the virus to spread into immunologically naïve communities. Additional vigilance should be taken in the absence of improved controls to identify such events. If on-arrival quarantine is expected to be used for a long time, quarantine facilities could be purpose-built to minimise the risk of transmission.

**Funding:**

10.13039/501100005847Health and Medical Research Fund, Hong Kong.

**Research in context**

**Evidence before this study**

On-arrival hotel quarantine has been one of the primary measures to prevent the introduction of SARS-CoV-2 into Hong Kong and many other countries since the start of the pandemic in 2020. However, hotels represent an ad hoc solution for quarantine as they are not necessarily designed for infection control. An in-depth analysis of SARS-CoV-2 transmissions in quarantine hotels is needed to help quantify the potential incidence of infection and promote awareness of the associated risk. We searched PubMed for studies published before 31 March 2022 reporting investigations of SARS-CoV-2 transmission within hotel quarantine, combining keywords “SARS-CoV-2” or “COVID-19” and “Quarantine” or “Isolation” or “Hotel”. Among the 1316 matching studies after removing duplicates, nine reported investigations of within-hotel transmission between guests and/or staff of SARS-CoV-2; five combined both genomic and epidemiologic data. One epidemiological study estimated a quarantine failure rate of 5.0 per 100,000 arrivals from 32 detected failures defined as an infection of staff or others linked to quarantine but not persons infected in quarantine. There have also been numerous reports of possible within-hotel transmission reported within the grey literature, including by press release, though not formally published as research.

**Added value of this study**

We investigate instances of within-hotel quarantine transmission in Hong Kong over a nearly two-year period using multiple streams of evidence, including genomics. This includes the identification and reclassification of ‘silent’ infectees that were detected whilst still in quarantine and therefore presumed to be infected overseas. This allows us to estimate the rate of within-hotel quarantine transmission among arrivals in Hong Kong. We report seven clusters of within-hotel transmission supported by genomic and epidemiologic data, of which five were not recognised at the time and presumed to be infected overseas.

**Implications of all the available evidence**

Together with previous reports, our findings characterise the low but non-zero risk of within-hotel transmission between unlinked guests in hotel quarantine despite strict infection-prevention controls in place, like those used in Hong Kong. In future pandemics, on-arrival quarantine in hotels could be used to delay the introduction of novel infections into a community. However, constructing purpose-built facilities for on-arrival quarantine may further reduce the marginal risk from importation.

## Introduction

One of the measures used to reduce the importation of COVID-19 into a locality is the quarantine of arriving persons. In Hong Kong, quarantine of arrivals at home, within purpose-built quarantine facilities or hotel quarantine (HQ), has been variably implemented since 27 January 2020, with mandatory quarantine for all arrivals since 19 March 2020. Quarantine within hotels for 14 days became mandatory for arrivals from some locations on 25 July 2020 and all arrivals from 13 November 2020, with few exceptions. From 22 December 2020 onwards, the Hong Kong government issued a list of hotels designated to receive inbound travellers, termed “designated hotel quarantine” (DHQ) which implemented increasingly stringent infection control measures.^1^^,^^2^ The required quarantine period also increased to a maximum of 21 days from 25 December 2020 as part of the response to the emergence of SARS-CoV-2 variants. However, it has sometimes varied depending on the global situation. Note that from this point onwards, we use HQ to include both terms HQ and DHQ unless a distinction between the two periods is necessary.

With a range of local control measures, importation control has resulted in the relatively limited local circulation of SARS-CoV-2 in Hong Kong in the first two years of the pandemic. By 31 January 2022, there had been approximately 13,000 confirmed cases, corresponding to just 1.9 confirmed cases per 1000 population in four distinct epidemic waves.^3^^,^^4^ However, in January 2022, the spread of Omicron BA.2 occurred in Hong Kong and was not controlled, resulting in a sizeable fifth epidemic wave with more than 1.1 million confirmed cases and more than 9000 deaths.^5^ A few infections have occasionally been traced back to infections between guests or staff working within HQ in Hong Kong.^6^ Transmission of infection from quarantined guests to other guests or staff has also been reported elsewhere and is now well-documented. Nine studies^7^, ^8^, ^9^, ^10^, ^11^, ^12^, ^13^, ^14^, ^15^ have reported investigations of within-hotel transmission of SARS-CoV-2 between guests and/or staff, though most were conducted to investigate individual outbreaks where suspected transmission had occurred, and only five combined both genomic and epidemiologic data in their investigation.^7^, ^8^, ^9^, ^10^, ^11^ Regular testing of hotel guests and staff can help to identify infections more quickly and limit leaks into the community. However, it can be challenging to identify transmission between quarantined persons because those who test positive in HQ would typically be assumed to have acquired their infection before they entered quarantine and therefore go unrecognised. In this study, we combined genomic and epidemiological data to investigate the risk of acquiring SARS-CoV-2 infection within HQ from 1 May 2020 to 31 January 2022 in Hong Kong.

## Methods

### Data collection and background

Line lists, including demographic data, were provided by the Centre for Health Protection (CHP) of the Department of Health for all cases confirmed by real-time-polymerase chain reaction (RT-PCR) in Hong Kong from 1 May 2020 through to 31 January 2022. Cases were characterised as “imported” if they had a travel history overseas during the 21-days prior to diagnosis and were presumed to have acquired the infection outside of Hong Kong (i.e., with no reason to presume otherwise). According to World Bank Development indicators, arrivals were classified into seven regions, given the most recent country of departure.

During the study period (1 May 2020–31 January 2022), all persons arriving in Hong Kong were tested upon arrival and then during quarantine (if negative at arrival) by RT-PCR. During the HQ period (1 May 2020–21 December 2020), guests were tested once before release towards the end of a 14-day quarantine period. In contrast, during the DHQ period (22 December 2020–31 January 2022), guests were tested by PCR up to six times during quarantine. However, testing frequency varied for many reasons, including the length of quarantine, country of departure, and local testing constraints. All laboratory-confirmed cases, including asymptomatic and mild cases, were isolated in hospitals or specialised isolation facilities during both periods. Based on the moment of detection, cases were classified as detected on arrival and excluded or negative on arrival. Negative on-arrival cases were further subset into those proceeding to HQ and those with special quarantine arrangements other than HQ. We excluded those with special quarantine arrangements, such as home quarantine for low-risk arrivals before 13 November 2020 and purpose-built quarantine for high-risk arrivals, among other specialised but minority arrangements (e.g., pilots and diplomats).

### Characterisation of potential HQ clustering

For cases that were negative by PCR on arrival and proceeded to HQ, we obtained the period of stay using the date of international arrival in Hong Kong as a proxy for arrival in HQ, and the date of admission to isolation facilities, indicating transfer from HQ. Though the exact date of arrival in HQ was unknown, in general, the median reported airport transit time was approximately 3–4 h, though the total time could be up to 12 h.^16^ Further, when measuring quarantine length in Hong Kong, the government counted the date of international arrival as day one and not the date of arrival in HQ in case of delay in transiting at the airport. All arrivals were temperature screened to detect fever and required to complete a health declaration reporting any respiratory symptoms, including cough, sore throat, difficulty breathing and shortness of breath. Those with symptoms were separated and referred to the Department of Health for corresponding arrangements, which at various times included isolation in a public hospital or dedicated isolation facility while waiting for confirmation by RT-PCR. Arrivals without symptoms were required to sit individually at chairs/desks physically distanced within a restricted area inside the open airport terminal while waiting for RT-PCR test results.^17^^,^^18^ At various times, arrivals were segregated depending on the varying risk levels depending on their country of departure.^19^ Mask-wearing throughout the arrival process was mandatory, with transit areas and surfaces regularly cleaned between arrivals. We have included a copy of a public infographic describing the arrival procedure and transfer to hotel quarantine for asymptomatic and RT-PCR-negative arrivals in Supplementary Fig. S1.

Where two or more guests were simultaneously confirmed for SARS-CoV-2 infection by RT-PCR and had an overlap in stay of one or more days within the same quarantine hotel but without reported close contact, i.e., staying in separate rooms, we undertook phylogenomic investigation to determine if the acquisition could have occurred within quarantine. Cases with known contact (e.g. family members that travelled together) were not considered in our analysis of within-HQ transmission because we aimed to focus on transmission between rooms rather than within the same room. For larger potential clusters, we did not require complete overlap-of-stay with all other cases, so long as a continuous overlap in stay could be traced between the group of cases. For example, the date of transfer from HQ for one case could be earlier than the date of arrival of another case in the cluster so long as a third intermediate case overlapped with both.

### Genomic sequencing and phylogenetic analysis

Saliva samples or nasopharyngeal swab samples of SARS-CoV-2 cases were sequenced. We reverse transcribed virus with primers targeting different regions with synthesised cDNA, then subjected to multiple overlapping 2-kb PCRs for full-genome amplification. We pooled PCR amplicons from identical specimens and sequenced them using the Nova sequencing platform (PE150, Illumina). Nextera XT prepared the sequencing library. Using Bcl2Fastq (Illumina), base-calling of raw read signal and demultiplexing of reads by different samples were performed. A reference-based re-sequencing strategy was applied in analysing the NGS data. Raw FASTQ reads were assembled and mapped to the SARS-CoV-2 reference genome (Wuhan-Hu-1, GenBank: MN90894 7.3) using BWA mem2 (v. 2.0pre2). We trimmed the primer and low-quality reads using iVar with the above primers and default parameters.^20^ The consensus sequences for each sample were called as dominant bases at each position by samtools mpileup (v. 1.11) and iVar with default parameters.^21^ Samples less than 27 kb in length (excluding gaps) were excluded from further downstream analysis.

We selected 350 available genome sequences for analysis given the criteria of epidemiological overlap defined previously. We aligned genomes with greater than 70% unambiguous nucleotides (n = 280) against A and B lineage references strains Wuhan/WH04/2020 and Wuhan-Hu-1/2019, respectively, using MAFFT v 7.49 for closely related viral genomes (FFT-NS-2 algorithm).^22^ Maximum likelihood phylogenetic trees were generated from the included alignment using IQ-Tree v 2.1.2 with 1000 ultrafast bootstrap replicates.^23^^,^^24^ We inferred sequence relatedness as the pairwise genetic distance between two sequences, calculating the number of genome-wide substitutions (HKY85 substitution model) given a total alignment length of 29,924 base pairs. Lineage assignment was determined using PANGOLIN v. 3.1.14 and PLEARN model v. 1.2.81, including the designation of variants of concern (VOC).^25^

### Analysis of potential HQ clustering

HQ transmission was determined between pairs identified by our initial screen using a varying threshold of genome-wise substitutions. For pairs with the same country of departure but very different arrival dates, transmission was considered probable if sequences were identical, i.e., zero genome-wise substitutions. This threshold was applied because we expect genetically similar viruses to circulate within the same departure country in time and thus exclude potential unlinked transmission before they arrive in Hong Kong as much as possible (Supplementary Fig. S2). For pairs reporting different countries of departure and different arrival dates, the criterion for probable transmission was relaxed to ≤3 genome-wide substitutions. The cut-off of ≤3 was determined based on the observed median genetic distance between known close contacts (e.g., families) also identified in HQ as we expect close contacts to be infected with similar or identical viruses. We observe this in Supplementary Fig. S2, which shows the comparatively broader genetic distance between all cases examined for potential HQ transmission, demonstrating the strictness of our genomic criteria. Regardless of overlap in HQ and genomic evidence, pairs with the same arrival date in Hong Kong and country of departure were not considered within-HQ transmissions because infection prior to entering HQ could not be excluded. For example, potential transmission could occur while waiting in the airport upon arrival or sharing designated transport to the hotels despite strict distancing measures, as shown in Supplementary Fig. S1. Table 1 summarises the criteria and level of evidence of within-HQ transmission.

**Table 1:** Criteria and evidence level used to evaluate clusters of potential within-HQ transmission between unliked guests.

Country of departure	Difference in arrival (days)	Genomically identical (0 subs)	Genomically similar (≤3 subs)
Same	0	–	–
	1	–	–
	2+	Probable	Plausible
Different	0	–	–
	1	Probable	Plausible
	2+	Probable	Probable

For all cases of potential within-HQ transmission, there must first be temporal and spatial overlap with other cases detected within the same hotel with or without available sequences.

Lastly, we calculate individual rates of HQ-acquired infection per positive case in HQ and per arrival, both positive and negative, required to quarantine in hotels. We use the number of probable and plausible within-HQ transmissions identified here, combined with all other within-HQ transmissions reported during the study period.

### Role of the funding source

This project was supported by the Health and Medical Research Fund, Food and Health Bureau, Government of the Hong Kong Special Administrative Region (grant no. COVID190205 & COVID190118). Funders had no role in the study design, the collection, analysis, and interpretation of data, the writing of the report, and the decision to submit the paper for publication. Ethics approval (UW 20-168) was obtained from the University of Hong Kong Institutional Review Board.

## Results

### Epidemiology of imported SARS-CoV-2 cases & sequence availability

During the study period, 13,165 cases were confirmed in Hong Kong, of which 23.9% (n = 3152/13,165) had been classified as imported, i.e., presumed to have acquired infection whilst overseas, while the remaining 76.1% (n = 10,013/13,165) of cases were determined to have acquired COVID-19 infection locally. Most imported cases arrived from South Asia, East Asia, or the Pacific, with detailed demographics in Supplementary Table S1.

**Fig. 1:**
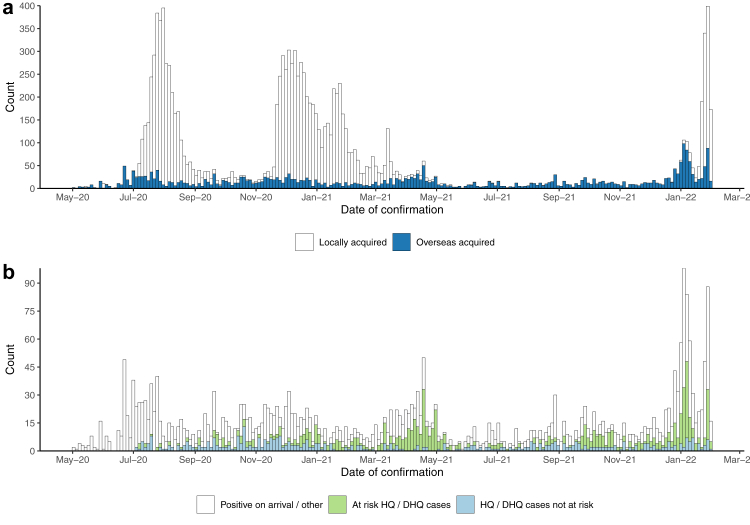
**a)** Epidemic curve of all cases of COVID-19 confirmed within Hong Kong during the study period (1 May 2020–31 January 2022) by the classified source of infection. **b)** Epidemic curve of all cases classified as overseas acquired subset into HQ and Non-HQ cases (positive on-arrival or not in HQ) and within overlapping stays in HQ with other cases in time and space.

Imported SARS-CoV-2 cases averaged 143 cases per month (Fig. 1) and peaked at 450 cases during January 2022 following the global emergence of the Omicron variant. The average monthly positivity rate of all overseas arrivals entering Hong Kong during the study period (n = 461,856) was 0.7% (Supplementary Fig. S3). Most cases classified as overseas acquired (62.3%, n = 1961/3152) did not stay in HQ but instead tested positive upon arrival at the airport and were sent to a specialised isolation facility or underwent quarantine other than HQ, e.g., home quarantine. The remaining 37.8% (n = 1189/3152) of cases classified as overseas acquired tested negative by PCR on arrival and proceeded to HQ, where they tested positive, 60.6% (n = 720/1189) of those had epidemiological overlap with other cases in HQ.

### Probable and plausible within HQ clusters

We identified 148 possible clusters among the 720 cases with epidemiological overlap detected in HQ which proceeded for genomic analysis. Valid genomic samples (>70% unambiguous nucleotides), however, were only available for 38.9% (n = 280/720) of those cases corresponding to 95 (64.2%, n = 95/148) possible clusters. However, 46 of those clusters had only one valid genome sample insufficient for comparative analysis or comprised only samples of known contacts travelling together and therefore were excluded.

**Fig. 2:**
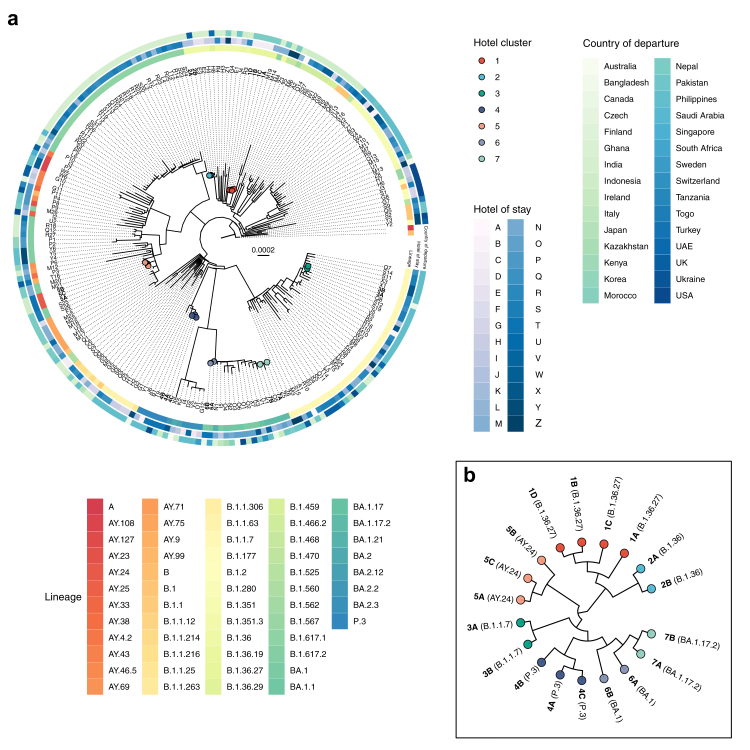
**a)** Phylogenetic tree of (n = 221) all available cases of SARS-CoV-2 confirmed within hotel quarantine (HQ) or designated hotel quarantine (DHQ) with overlap to other unlinked cases in Hong Kong from 1 May 2020 through 31 January 2022 aligned against representative sequences of SARS-CoV-2 lineages A and B. Tips are coloured as confirmed within-HQ transmission clusters and labelled with unique case identifiers equivalent to Fig. 3. Non HQ cluster cases are labelled with unique identifiers equivalent to the hotel of stay. The radial heat map shows PANGO lineage, hotel of stay, and country of departure. **b)** Cladogram of suspected HQ clusters.

**Fig. 3:**
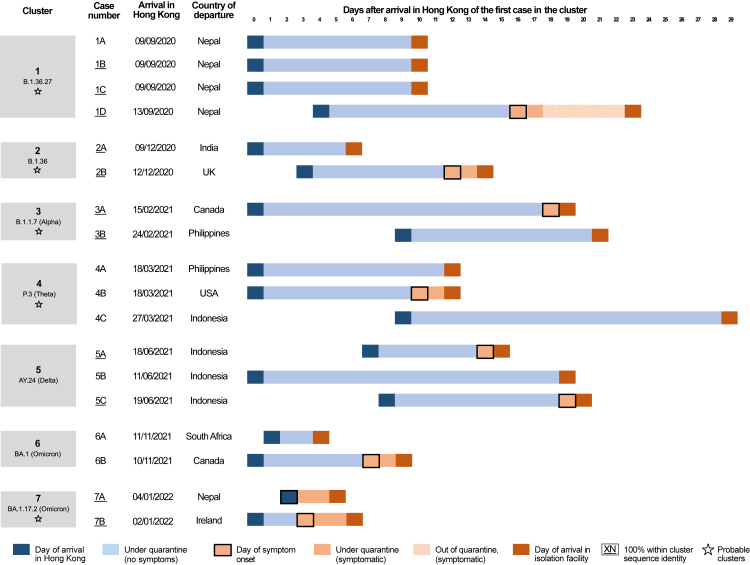
Cases of confirmed SARS-CoV-2 transmission between epidemiologically unlinked arrivals within hotel quarantine (HQ) or designated hotel quarantine (DHQ) in Hong Kong from 1 May 2020 through 31 January 2022. Case identifiers in each cluster are ordered by reporting date (Supplementary Table S6).

Of the remaining 49 clusters, genomic analysis initially identified 12 clusters of possible within-HQ transmission. However, five of these clusters were discarded because infection before entering HQ (e.g., waiting at the airport on arrival or shared transport to the hotels) was equally likely, resulting in seven within HQ clusters (Figs. 2 and 3), comprising five probable clusters and two plausible clusters.

Cluster one comprised four cases from Nepal in September 2020, including three family contacts (cases 1A, 1B, 1C) arriving on the same day, while the unlinked case 1D arrived in Hong Kong four days later. All four cases belonged to lineage B.1.36.27. This probable HQ cluster is determined by the observation that case 1D shared 100% pairwise sequence identity with cases 1B and 1C, and the probable source of infection, case 1A, was phylogenetically basal to the three identical cases (Fig. 2). This conclusion is also supported by the epidemiological information that case 1D developed symptoms following completion of quarantine after testing negative on day 13 of 14. Case 1D was subsequently linked as the source of infection for three local relatives, likely initiating the fourth epidemic wave of COVID-19 in Hong Kong.^26^

Probable HQ cluster two included cases 2A & 2B. Case 2A arrived in Hong Kong from India in December 2020, and case 2B arrived from the UK three days later. Despite different countries of departure and arrival dates, each shared 100% pairwise sequence identity and belonged to lineage B.1.36. The direction of transmission could not be inferred because sequences were identical. However, the overlap from arrival in Hong Kong to confirmation or onset between the two cases suggests that case 2A was the likely infector of case 2B (Fig. 3).

Probable cluster three (contemporaneously investigated and reported by local health officials at the time) involved cases 3A & 3B. Case 3A arrived from Canada in February 2021, while case 3B arrived in Hong Kong from the Philippines nine days later. Both were required to undergo 21-day mandatory quarantine due to a policy change (effective from 2 February 2021). As with probable cluster two, despite different countries of departure and arrival dates, both cases shared 100% pairwise sequence identity and belonged to lineage B.1.1.7 (Alpha VOC). Given the epidemiological overlap (Fig. 3) and the latent period distribution of SARS-CoV-2 (Median: 5.5 days, upper 95th percentile: 10.6 days^27^), it is more likely that 3B infected 3A.

Probable cluster four involved three cases: 4A, 4B and 4C. Cases 4A and 4B arrived in Hong Kong on the same day in March 2021, from the Philippines and the USA, respectively, and were required to undergo 21 days quarantine. Both sequences were separated by two genome-wise substitutions and belonged to lineage P.3 (Theta VOC) despite different countries of departure (Figs. 2 and 3). The third case, 4C, arrived in Hong Kong nine days after 4A and 4B from Indonesia and was separated by two and three genome-wise substitutions from the two cases, respectively. We cannot exclude the possibility of transmission between 4A and 4B occurring before entering HQ given the lack of information on shared transport and transit times within the Hong Kong airport. However, transmission between 4A/4B and 4C who arrived days later was considered probable.

Plausible cluster five comprised three cases: 5A, 5B, and 5C who arrived in Hong Kong from Indonesia, with 5B arriving first, 5A seven days later, and 5C eight days later than 5B. 5A and 5C shared 100% sequence identity, but were not considered plausible becasue they arrived from Indonesia within one-day of each other (Table 1, Fig. 3). HQ transmission was considered plausible in 5B who was separated by two substitutions from the other two cases but arrived seven to eight days before the others. All three belonged to lineage AY.24, a Delta-like (B.1.617.2) subvariant (Fig. 2). Given the dates of arrival and symptom onset, it is possible that case 5B might be the asymptomatic infector of both 5A and 5C (Fig. 3).

The last two HQ clusters (six & seven) each comprised two sequenced cases and belonged to Omicron variant lineages or sublineages of BA.1. The two cases, 6A and 6B, from the cluster six (contemporaneously reported, BA.1 Omicron lineage) arrived from South Africa and Canada one day apart in November 2021 with their sequences differed by only one nucleotide substitution.^7^ A minor possibility of pre-HQ transmission could not be excluded due to the potential for extended transit through the Hong Kong airport, given their close arrival date. The two cases of the seventh and final cluster (BA.1.17.2), 7A and 7B, arriving two days apart from Nepal and Ireland in January 2022 and shared identical genomic sequences indicating probable within-HQ transmission.

### Risk of within HQ transmission & sensitivity analysis

Among the 221 sequenced cases with overlapped stay with other case(s) in the same hotel, 158 were positive for a VOC, the most common being Delta (B.1.617.2-like, n = 42/158, 27%) and Alpha (B.1.1.7-like, n = 38/158, 24%). However, there was no evidence that sequenced HQ transmission clusters were more likely to involve a VOC compared to other cases detected in HQ though the case numbers within HQ clusters were small (OR = 0.78, 95% CI: 0.29–2.33, p = 0.64, Logistic regression, Supplementary Table S2). Likewise, potential risk factors associated with suspected within-HQ transmission, i.e., longer arrival-to-onset and arrival-to-confirmation intervals for asymptomatic cases, was not significantly associated with probable and plausible HQ transmission clustering (OR = 1.14, 95% CI: 0.98–1.35, p = 0.09 & OR = 1.11, 95% CI: 0.98–1.25, p = 0.08, logistic regression, arrival-to-onset & report respectively, Supplementary Table S3). However, longer intervals were significantly associated with cases sequenced and included for analysis (n = 221) compared to those missing sequences and/or sequenced but excluded cases (Supplementary Table S4) indicating potential selection bias in favour of within-HQ cluster cases. As such, we can assume that rate of probable and plausible clustering among the cases identified in HQ, but missing sequence data, was lower than sequenced and included cases.

From our sensitivity analysis given the five probable (pre-HQ transmission excluded) and two plausible HQ clusters identified (conservatively assuming only one HQ infectee per cluster), we estimate a minimum of 4–6 cases for every 1000 identified in HQ (n = 1189 HQ cases) could have been infected within HQ rather overseas, and a maximum expected rate of 13–18 cases per 1000 (Supplementary Table S5). This excludes those likely infected by a known contact staying in the same room or travelling together, e.g., family members. We also estimate that among all inbound arrivals sent to HQ (positive and negative), a minimum of 1–2 per 100,000 could be infected during HQ rather than overseas, and a maximum of 3–5 per 100,000 (Supplementary Table S5).

## Discussion

In this study, we present the descriptive epidemiology of all COVID-19 cases imported into Hong Kong between 1 May 2020 and 31 January 2022 and reported plausible SARS-CoV-2 transmission between persons without apparent contact other than a temporally overlapped stay in the same hotel for quarantine. We identified seven clusters of within-HQ transmission, five of which were unrecognised at the time among the 49 clusters with available genomic data. We consider five of the seven clusters as ‘probable' after excluding all reasonable transmission avenues before entering HQ. In contrast, the remaining two were considered ‘plausible’ given the chance, though unlikely, of infection within common countries of departure (cluster five) or transmission between arrival in Hong Kong and entering HQ (cluster six).

Beyond the 5–7 clusters reported in our study, five additional instances of probable within-HQ transmission, and one plausible instance, have been investigated and reported by the Centre for Health Protection in Hong Kong during our study period.^2^^,^^28^, ^29^, ^30^, ^31^, ^32^, ^33^ This brings the total number of observed cases likely infected within HQ in Hong Kong to 10–13. When accounting for these additional observed clusters outside the ones we have identified here, we estimate that per 1000 cases identified in HQ, 8–11 could have been infected within HQ rather than overseas. Likewise, when accounting for the additional reporting of observed clusters, we estimate that 2–3 per 100,000 arrivals sent to HQ (n = 457,932) could be infected within HQ, an increase of 1–2 per 100,000 from our data alone, thus characterising the low but non-zero chance of within-hotel transmission between unlinked travellers in HQ. One previous study reported a quarantine failure rate of 5 per 100,000 arrivals though this only considered infection of quarantine staff and other persons linked to the quarantine system but not transmission between arrivals within HQ which suggests quarantine staff may be at a higher risk of infection from guests than between guests.^13^

Despite this low chance, a within-HQ transmission event can have high consequences. For example, a single instance of probable within-HQ transmission reported in this study (cluster one) was later determined to be responsible for the introduction of B.1.36.27 into Hong Kong, which initiated the fourth epidemic wave and resulted in widespread infection and substantial economic impacts as public health measures mandated business closures. Similarly, Hong Kong's fifth and largest COVID-19 wave was triggered by separate HQ transmissions of BA.2 outside the study period.^33^ This wave comprised more than 1.1 million cases and over 9000 deaths,^34^ demonstrating the potentially severe consequences of low-frequency HQ transmission events. Additionally, there have been other introductions of novel lineages in Hong Kong where the source could not be identified despite strict quarantine measures, including the unidentified introduction(s) which resulted in the third epidemic wave in Hong Kong.^3^

Our study has a few limitations. Firstly, valid samples or full-length sequence data were only available for a minority of cases identified as potential episodes of HQ transmission. Further, among the potential HQ clusters with available sequence data, roughly half were excluded because they comprised cases with only one sample or samples of known family contacts. We also identified significantly longer arrival-to-onset and arrival-to-confirmation intervals among sequenced cases compared to unsequenced and excluded cases, which indicates potential selection bias and sequencing effort favouring cases more likely to be infected in HQ and would likely result in an overestimation of the overall risk. We were, however, especially cautious not to extrapolate the observed rate of HQ clustering to those clusters with missing data and instead present only those observed (both reported in our study and elsewhere). We do expect, however, there may be additional clusters among the missing data that have gone unrecognised. For example, low Ct-values and abnormally long incubation periods indicative of recent infection have been reported in other studies in Hong Kong among cases detected in HQ beyond those reported here.^35^

Second, data on potential contact or proximity of unlinked HQ cases such as room or floor of stay within HQ was not readily available. However, the proximity of hotel rooms in Hong Kong has been noted among some of the probable clusters reported outside our study following government investigations.^2^ Beyond proximity, other risk factors noted in Hong Kong included opening external windows of rooms (potential for air draft towards hallways and other rooms), guests exercising (increased exhalation), the meal delivery process (food hooks), and lack of mask-wearing by quarantined persons whilst inside their quarantine room as recommended.^2^^,^^28^, ^29^, ^30^, ^31^, ^32^, ^33^ After each investigation, changes were made to HQ guidelines for hotel management and guests (Supplementary Fig. S4), such as requiring that windows remain closed and the provision of air purifiers for those wishing to exercise. Outside of Hong Kong, opening doors for testing or meal collection have also been noted as common risk factors associated with HQ transmission despite strict protocols to reduce such risks.^9^^,^^11^ In our study, data on proximity or other risk factors could have provided additional evidence to support identified instances of HQ transmission or support excluded instances lacking genomic data.

There was also no data on indirect contact between unlinked cases before arriving in Hong Kong, such as shared time within the airport. Though we tried to control for this by excluding clusters with identical arrival dates,^36^ the potential for prolonged airport transit was possible and noted in at least one case (cluster six). Despite this, our criteria for probable and plausible clustering were quite strict, combining multiple sources of evidence and reporting only clusters where pre-HQ transmission was excluded or reasonably unlikely. Furthermore, the lower bound of our estimates only counts the five most probable HQ transmission clusters (Fig. 3). We stress however that our estimates calculated here may not be generalisable to other localities due to dependence on the varied factors associated with the risk of HQ transmission. For example, the number of quarantine hotels available, including capacity limitations, the strictness of infection control measures, and the overall positivity rate among HQ arrivals, which may vary significantly between localities. The rates presented here also do not include staff infected within HQ, which, though a separate phenomenon, has also been reported in Hong Kong^6^ and should therefore not be extrapolated from our results. Future studies could look to sequence all SARS-CoV-2 cases detected in HQ (including staff) to estimate the underlying rate of HQ transmission. The relative effects of potential risk-mitigation factors such as ventilation, room spacing, and HQ capacity could then also be measured.

In conclusion, we report at least seven instances of within-HQ transmission in Hong Kong, five of which were considered probable among the five to six others clusters not counted here but reported in Hong Kong within the study period. We have systematically demonstrated the risk of within-HQ transmission associated with confining travellers to HQ and identified a low but non-zero rate of SARS-CoV-2 transmission between individuals in HQ without direct contact. In the event of a future pandemic, governments aiming to prevent or delay local outbreaks should be aware of the non-zero risk of within-HQ transmission and weigh the consequence of community introductions of pathogenic agents against the ongoing economic and social impacts of such measures. In the meantime, HQ infection prevention and control measures should be reviewed for potential improvements, including quantifying associated risk factors. Alternatively, the use of robust purpose-built quarantine facilities could also be considered when a novel or emerging pathogen is perceived with severe public health consequences.

## Contributors

All authors meet the ICMJE criteria for authorship. L.L.M.P. and B.J.C. conceived the study. H.G. analysed the NGS data. D.C.A. wrote the first draft of the manuscript and conducted the epidemiological and genomic analyses. M.M.-S., B.Y., and Y.L. compiled the epidemiological dataset and M.M.-S. assisted in writing the first draft. All authors provided critical interpretation, review, and revision of the text, and approved the final version.

## Data sharing statement

All final genomic sequences are made available online upon publication via GISAID and via https://github.com/dcadam/covid-19-dhq. All individual demographic data of sequenced cases also used in this study after de-identification are available in Supplementary Table S6.

## Declaration of interests

B.J.C. consults for AstraZeneca, Fosun Pharma, GlaxoSmithKline, Moderna, Pfizer, Roche and Sanofi Pasteur. The authors report no other potential conflicts of interest.
