# Supplementary material for: Risk of within-hotel transmission of SARS-CoV-2 during on-arrival quarantine in Hong Kong: an epidemiological and phylogenomic investigation
Source: Lancet Reg Health West Pac. 2023 Jan 7;33:100678. doi: 10.1016/j.lanwpc.2022.100678 (PMC9825110; doi:10.1016/j.lanwpc.2022.100678)
Supplement: Supplementary Tables S1–S6 and Figures S1–S4 [file mmc1.docx]

**Supplementary Material**

**Supplementary Figure S1.** Infographic of the airport arrival process in Hong Kong provided by the Hong Kong Government to travellers.

**Supplementary Figure S2.** Comparison of pair-wise genetic distance calculated as the number of substitutions between paired cases detected in HQ coloured by category of epidemiological overlap.

**Supplementary Figure S3.** Number of persons arriving in Hong Kong via the international airport by month of arrival and COVID-19 test result during the study period (1 May 2020 to 31 Jan 2022).

**Supplementary Figure S4.** Example infographic provided in hotel quarantine to guest to reduce the risk of transmission between guests.

**Supplementary Table S1.** Descriptive epidemiology of SARS-CoV-2 cases confirmed in Hong Kong and characterised as overseas acquired cases from 1 May 2020 – 31 January 2022

**Supplementary Table S2**. SARS-CoV-2 variants of concern (VOC) identified among cases identified within DHQ transmission clusters and non-DHQ cluster cases.

**Supplementary Table S3**. Results of logistic regression analysis between arrival-to-onset and arrival-to-confirmation interval in days among sequenced cases (n=221 and evidence of probable and plausible within HQ transmission clustering.

**Supplementary Table S4**. Results of logistic regression analysis between arrival-to-onset and arrival-to-confirmation interval in days and sequence availability or exclusion in the clustering analysis.

**Supplementary Table S5**. Sensitivity analysis for the expected number of within-HQ clusters and infection rates per 1000 cases detected in HQ and per 100,000 overseas arrivals required to quarantine.

**Supplementary Table S6**. Travel related SARS-CoV-2 cases detected in designated hotel quarantine in Hong Kong between 1 May 2020 and 31 Jan 2022 with available sequence data linked into at risk epidemiological clusters based on hotel of stay and dates or arrival into Hong Kong and admission to isolation facility.


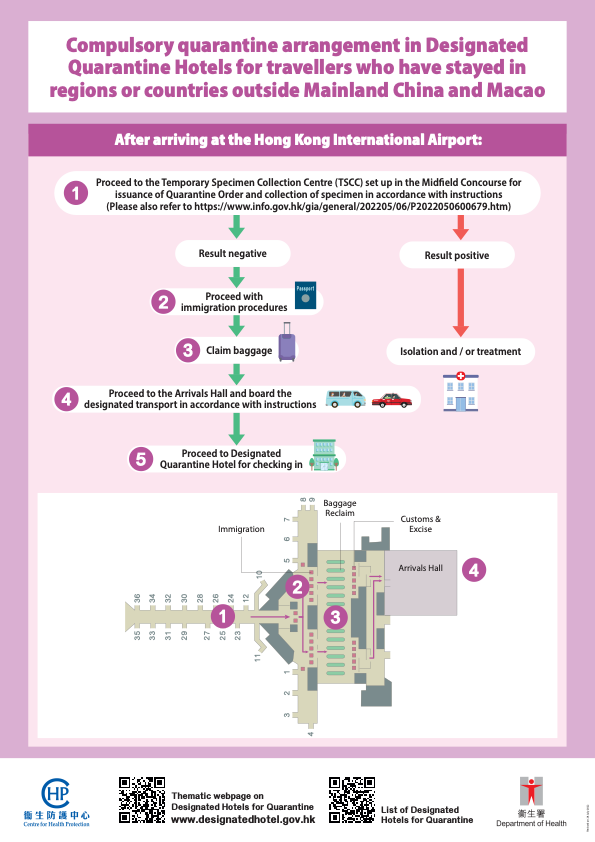
**Supplementary Figure S1.** Infographic of the airport arrival process in Hong Kong provided by the Hong Kong Government to travellers. Strict physical distancing is enforced during transit, including whilst waiting for on arrival results, and on dedicated shuttles for each hotel which run at half capacity.

**Supplementary Figure S2.** Comparison of pair-wise genetic distance calculated as the number of substitutions between paired cases detected in HQ coloured by category of epidemiological overlap. Panels are separated into pairs with and without evidence of common departure i.e. those with the same year-month of arrival and country of departure. The median genetic distance between pairs linked as known close contacts (e.g. family members) was used to define the maximum threshold criterion to identify likely HQ transmissions, including the exclusion of potential pre-HQ transmission as described in the methods.

**Supplementary Figure S3.** Number of persons arriving in Hong Kong via the international airport by month of arrival and COVID-19 test result during the study period (1 May 2020 to 31 Jan 2022). Positive arrivals (n=3,152) include those who tested positive on arrival, in hotel quarantine, and post-quarantine but suspected to have acquired infection overseas or within quarantine. The number of negative arrivals (n=458,708) was calculated as the difference between the reported total persons (n=461,856) arriving each month and those testing positive. Numbers are presented on a log-scale due to the relatively low number of positive arrivals. The line and points represent the percent of total positive arrivals by month. The mean positivity of arrivals during the study period was 0.7%.


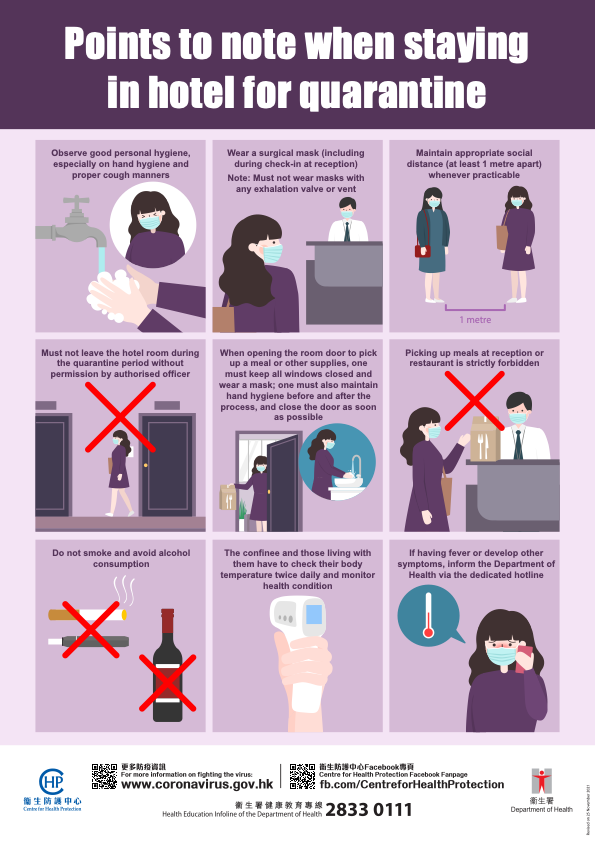


**Supplementary Figure S4.** Example infographic provided in hotel quarantine to guest to reduce the risk of transmission between guests.

**Supplementary Table S1.** Descriptive epidemiology of SARS-CoV-2 cases confirmed in Hong Kong and characterised as overseas acquired cases from 1 May 2020 – 31 January 2022

|  |  | HQ with overlap | | HQ cases | Non-HQ cases | Total Pos. Imported  (n=3,152) |
| --- | --- | --- | --- | --- | --- | --- |
| Descriptive variables | | Seqs  (n=221) | Total  (n=720) | Total  (n=1,189) | Total  (n=1,963) |  |
| *Sex* | |  |  |  |  |  |
|  | Female | 128 (57.9%) | 445 (61.9%) | 659 (55.4%) | 985 (50.2%) | 1644 (52.2%) |
|  | Male | 93 (42.1%) | 275 (38.1%) | 530 (44.6%) | 978 (49.8%) | 1508 (47.8%) |
| *Age* | |  |  |  |  |  |
|  | 0-19 | 31 (14.0%) | 87 (12.1%) | 151 (12.7%) | 255 (13.0%) | 406 (12.9%) |
|  | 20-39 | 92 (41.6%) | 376 (52.3%) | 598 (50.3%) | 1036 (52.8%) | 1634 (51.8%) |
|  | 40-59 | 78 (35.3%) | 200 (27.8%) | 354 (29.8%) | 546 (27.8%) | 900 (28.6%) |
|  | 60-79 | 19 (8.6%) | 52 (7.1%) | 80 (6.7%) | 121 (6.2%) | 201 (6.4%) |
|  | 80+ | 1 (0.5%) | 5 (0.7%) | 6 (0.5%) | 5 (0.3%) | 11 (0.3%) |
|  |  |  |  |  |  |  |
| *Import region* | |  |  |  |  |  |
|  | East Asia & Pacific | 78 (35.3%) | 300 (41.6%) | 400 (33.6%) | 550 (28.0%) | 950 (30.1%) |
|  | Europe & Central Asia | 21 (9.5%) | 82 (11.4%) | 242 (20.4%) | 443 (22.6%) | 685 (21.7%) |
|  | Latin America & Caribbean | 0 (0.0%) | 2 (0.3%) | 7 (0.6%) | 26 (1.3%) | 33 (1.0%) |
|  | Middle East & North Africa | 4 (1.8%) | 20 (2.8%) | 46 (3.9%) | 71 (3.6%) | 117 (3.7%) |
|  | North America | 14 (6.3%) | 38 (5.3%) | 91 (7.7%) | 143 (7.3%) | 234 (7.5%) |
|  | South Asia | 97 (43.9%) | 257 (35.7%) | 357 (30.0%) | 672 (34.2%) | 1029 (32.7%) |
|  | Sub-Saharan Africa | 5 (2.3%) | 16 (2.2%) | 35 (2.9%) | 41 (2.1%) | 76 (2.4%) |
|  | Missing | 2 (0.9%) | 5 (0.7%) | 11 (0.9%) | 17 (0.9%) | 28 (0.8%) |

**Supplementary Table S2**. SARS-CoV-2 variants of concern (VOC) identified among cases identified within DHQ transmission clusters and non-DHQ cluster cases. The odds that a DHQ case was phylogenetically linked to a DHQ cluster involving a VOC was 0.78 (95% CI = 0.29, 2.33) though this was not statistically significant (p = 0.64, Logistic regression)

|  | DHQ  cluster cases | non-DHQ  cluster cases | Row  Total |
| --- | --- | --- | --- |
| VOC | 12 | 146 | 158 |
| non-VOC | 6 | 57 | 63 |
| Column Total | 18 | 203 | 221 |

**Supplementary Table S3**. Results of logistic regression analysis between arrival-to-onset and arrival-to-confirmation interval in days among sequenced cases (n=221 and evidence of probable and plausible within HQ transmission clustering. Results were not statistically significant.

| Variable | β | 95% CI | SE | p |
| --- | --- | --- | --- | --- |
| Arrival-to-onset | 0.13 | -0.02, 0.29 | 0.08 | 0.094 |
| Arrival-to-confirmation | 0.10 | -0.01, 0.22 | 0.06 | 0.087 |

**Supplementary Table S4**. Results of logistic regression analysis between arrival-to-onset and arrival-to-confirmation interval in days and sequence availability or exclusion in the clustering analysis. Sequenced cases had statistically longer arrival intervals compared to unsequenced cases, i.e. missing and/or excluded, indicating potential selection / sequencing bias favouring clustering.

| Sequenced | Variable | β | 95% CI | SE | p |
| --- | --- | --- | --- | --- | --- |
| 211/720 | Arrival-to-onset | 0.12 | 0.05, 0.20 | 0.04 | 0.001 |
|  | Arrival-to-confirmation | 0.03 | 0.003, 0.052 | 0.01 | 0.03 |
| 350/720 | Arrival-to-onset | 0.16 | 0.08, 0.25 | 0.04 | < 0.001 |
|  | Arrival-to-confirmation | 0.10 | 0.07, 0.13 | 0.02 | < 0.001 |

**Supplementary Table S5**. Sensitivity analysis for the expected number of within-HQ clusters and infection rates per 1000 cases detected in HQ and per 100,000 overseas arrivals required to quarantine. Counts and rates are calculated as the total of the observed and expected number of clusters. See the footnotes for examples.

| Percentage of observed rate for missing clusters | Obs + Exp clusters | Infection risk per 1000 cases | Infection risk per 100,000 arrivals |
| --- | --- | --- | --- |
| 0% | 5 - 7 | 4 - 6 | 1 - 2 |
| 10% | 6 - 8 | 5 - 7 | 1 - 2 |
| 20% | 7 - 10 | 6 - 8 | 2 - 2 |
| 30% | 8 - 11 | 7 - 9 | 2 - 2 |
| 40% | 9 - 13 | 8 - 11 | 2 - 3 |
| 50% | 10 - 14 | 8 - 12 | 2 - 3 |
| 60% | 11 - 15 | 9 - 13 | 2 - 3 |
| 70% | 12 - 17 | 10 - 14 | 3 - 4 |
| 80% | 13 - 18 | 11 - 15 | 3 - 4 |
| 90% | 14 - 20 | 12 - 17 | 3 - 4 |
| 100% | 15 - 21 | 13 - 18 | 3 - 5 |

The number of additional clusters expected is calculated as a proportion of the possible clusters with missing sequence data or those excluded from the final analysis (n=99) given an observation rate and the proportion of HQ clusters (n=5-7) identified among those included (n=49). For example, at 0% of the observed proportion, we expect no other clusters among the missing data, while at 40% we expect 4-6 additional but unobserved clusters among the missing data. If we assume the rate of HQ clustering was the same among the observed and missing data, we expect an additional 10-14 clusters or 15-21 total.

**Supplementary Table S6**. Travel related SARS-CoV-2 cases detected in designated hotel quarantine in Hong Kong between 1 May 2020 and 31 Jan 2022 with available sequence data linked into at risk epidemiological clusters based on hotel of stay and dates or arrival into Hong Kong and admission to isolation facility. Note that complete arrival dates were known for all cases but removed here to protect against re-identifiability where possible.

| Phylo. Cluster | Risk. Cluster | Case | Lineage | Variant | Departure | Hotel | Arrival | Arrival-to-onset | Arrival-to-isolation | Arrival-to-report |
| --- | --- | --- | --- | --- | --- | --- | --- | --- | --- | --- |
| 1A | 1 | C1 | B.1.36.27 |  | Nepal | C | Sep 2020 |  | 10 | 11 |
| 1B | 1 | C2 | B.1.36.27 |  | Nepal | C | Sep 2020 |  | 10 | 11 |
| 1C | 1 | C3 | B.1.36.27 |  | Nepal | C | Sep 2020 |  | 10 | 11 |
| 1D | 1 | C4 | B.1.36.27 |  | Nepal | C | Sep 2020 | 12 | 19 | 21 |
|  | 1 | C5 | B.1.560 |  | India | C | Sep 2020 |  | 10 | 11 |
|  | 1 | C6 | B.1.560 |  | India | C | Sep 2020 |  | 10 | 12 |
| 2A | 2 | J1 | B.1.36 |  | India | J | Dec 2020 |  | 6 | 8 |
| 2B | 2 | J2 | B.1.36 |  | United Kingdom | J | Dec 2020 | 9 | 11 | 12 |
|  | 2 | J3 | B.1.2 |  | USA | J | Dec 2020 | 1 | 6 | 7 |
|  | 2 | J4 | B.1.36.19 |  | Indonesia | J | Jan 2021 | 11 | 14 | 14 |
| 3A | 3 | R1 | B.1.1.7 | Alpha | Canada | R | Feb 2021 | 18 | 19 | 20 |
| 3B | 3 | R2 | B.1.1.7 | Alpha | Philippines | R | Feb 2021 |  | 12 | 13 |
|  | 3 | R5 | B.1.459 |  | Indonesia | R | Feb 2021 |  | 12 | 13 |
|  | 3 | R6 | B.1.466.2 |  | Indonesia | R | Feb 2021 |  | 12 | 13 |
| 4A | 4 | T6 | P.3 | Theta | Philippines | T | Mar 2021 |  | 12 | 13 |
| 4B | 4 | T7 | P.3 | Theta | USA | T | Mar 2021 | 10 | 12 | 13 |
| 4C | 4 | T8 | P.3 | Theta | Indonesia | T | Mar 2021 |  | 19 | 20 |
|  | 4 | T10 | B.1.351 | Beta | Philippines | T | Mar 2021 | 11 | 12 | 13 |
|  | 4 | T11 | B.1.617.1 | B.1.617.1-like | India | T | Apr 2021 |  | 13 | 14 |
|  | 4 | T12 | B.1.617.1 | B.1.617.1-like | India | T | Apr 2021 |  | 13 | 14 |
|  | 4 | T13 | B.1.617.1 | B.1.617.1-like | India | T | Apr 2021 |  | 14 | 14 |
|  | 4 | T14 | B.1.1.7 | Alpha | Pakistan | T | Apr 2021 |  | 13 | 13 |
|  | 4 | T15 | B.1.1.7 | Alpha | USA | T | Apr 2021 | 10 | 12 | 13 |
|  | 4 | T9 | P.3 | Theta | Philippines | T | Mar 2021 |  | 12 | 13 |
| 5A | 5 | T1 | AY.24 | Delta | Indonesia | T | Jun 2021 | 7 | 8 | 9 |
| 5B | 5 | T2 | AY.24 | Delta | Indonesia | T | Jun 2021 |  | 19 | 20 |
| 5C | 5 | T3 | AY.24 | Delta | Indonesia | T | Jun 2021 | 11 | 12 | 13 |
|  | 5 | T16 | AY.9 | Delta | United Kingdom | T | Jun 2021 | 3 | 3 | 4 |
| 6A | 6 | T4 | BA.1 | Omicron | South Africa | T | Nov 2021 |  | 3 | 4 |
| 6B | 6 | T5 | BA.1 | Omicron | Canada | T | Nov 2021 | 7 | 9 | 10 |
| 7A | 7 | M1 | BA.1.17.2 | Omicron | Nepal | M | Jan 2022 | 0 | 3 | 4 |
| 8B | 7 | M2 | BA.1.17.2 | Omicron | Ireland | M | Jan 2022 | 3 | 6 | 8 |
|  | 8 | D1 | BA.2.3 | Omicron | Philippines | D | Dec 2021 | 2 | 4 | 4 |
|  | 8 | D2 | BA.2.3 | Omicron | Philippines | D | Jan 2022 | 1 | 2 | 3 |
|  | 9 | B1 | P.3 | Theta | Philippines | B | Mar 2021 | 5 | 12 | 13 |
|  | 9 | B2 | B.1.1.7 | Alpha | Philippines | B | Mar 2021 | 11 | 13 | 13 |
|  | 9 | B3 | B.1.351 | Beta | Philippines | B | Mar 2021 |  | 13 | 13 |
|  | 10 | E1 | B.1.36 |  | Pakistan | E | Nov 2020 |  | 17 | 18 |
|  | 10 | E2 | B.1.36 |  | India | E | Dec 2020 |  | 13 | 14 |
|  | 10 | E3 | B.1.36 |  | India | E | Dec 2020 |  | 13 | 14 |
|  | 11 | G1 | B.1.1.63 |  | Philippines | G | Jul 2020 |  | 1 | 2 |
|  | 11 | G2 | B.1.1.63 |  | Philippines | G | Jul 2020 |  | 1 | 2 |
|  | 11 | G3 | B.1.1.263 |  | Philippines | G | Jul 2020 | 1 | 1 | 2 |
|  | 12 | G4 | B.1.36 |  | India | G | Jul 2020 |  | 2 | 1 |
|  | 12 | G5 | B.1.1.306 |  | India | G | Jul 2020 |  | 5 | 2 |
|  | 12 | G6 | B.1.1 |  | India | G | Jul 2020 |  | 5 | 1 |
|  | 13 | G7 | B.1.280 |  | USA | G | Dec 2020 | 9 | 12 | 13 |
|  | 13 | G8 | B.1.280 |  | USA | G | Dec 2020 | 4 | 12 | 13 |
|  | 13 | G9 | B.1.459 |  | Indonesia | G | Dec 2020 |  | 12 | 13 |
|  | 14 | H1 | B.1.1.63 |  | Philippines | H | Sep 2020 |  | 10 | 12 |
|  | 14 | H2 | B.1.1.63 |  | Philippines | H | Sep 2020 |  | 10 | 12 |
|  | 15 | H4 | B.1.36 |  | India | H | Dec 2020 | 11 | 12 | 13 |
|  | 15 | H5 | B.1.1.7 | Alpha | United Kingdom | H | Dec 2020 | 5 | 12 | 13 |
|  | 16 | H6 | B.1.567 |  | USA | H | Dec 2020 |  | 12 | 13 |
|  | 16 | H7 | B.1.567 |  | USA | H | Dec 2020 | 7 | 12 | 13 |
|  | 16 | H8 | B.1.36.29 |  | UAE | H | Jan 2021 | 6 | 14 | 14 |
|  | 17 | J5 | B.1.1.7 | Alpha | Philippines | J | Mar 2021 |  | 13 | 14 |
|  | 17 | J6 | P.3 | Theta | Philippines | J | Mar 2021 |  | 13 | 13 |
|  | 18 | M3 | B.1.1.7 | Alpha | United Kingdom | M | Dec 2020 | 9 | 10 | 12 |
|  | 18 | M4 | B.1.36 |  | Nepal | M | Dec 2020 |  | 13 | 13 |
|  | 18 | M5 | B.1.1.214 |  | Japan | M | Dec 2020 | 1 | 12 | 13 |
|  | 19 | M10 | B.1.1.7 | Alpha | Philippines | M | Mar 2021 |  | 12 | 13 |
|  | 19 | M11 | B.1.351 | Beta | Philippines | M | Mar 2021 |  | 12 | 13 |
|  | 19 | M6 | B.1.562 |  | India | M | Feb 2021 |  | 13 | 14 |
|  | 19 | M7 | B.1.351 | Beta | Philippines | M | Feb 2021 |  | 12 | 13 |
|  | 19 | M8 | B.1.1.7 | Alpha | UAE | M | Mar 2021 | 3 | 3 | 5 |
|  | 19 | M9 | B.1 |  | Indonesia | M | Mar 2021 | 11 | 12 | 13 |
|  | 20 | Q1 | B.1 |  | Pakistan | Q | Aug 2020 |  | 15 | 16 |
|  | 20 | Q2 | B.1.1.63 |  | Philippines | Q | Aug 2020 | 8 | 10 | 11 |
|  | 20 | Q3 | B.1.1.63 |  | Philippines | Q | Aug 2020 | 10 | 10 | 11 |
|  | 21 | Q4 | B.1.1.216 |  | Nepal | Q | Oct 2020 | 6 | 10 | 12 |
|  | 21 | Q5 | B.1 |  | Pakistan | Q | Oct 2020 |  | 10 | 11 |
|  | 22 | Q10 | B.1.617.1 | B.1.617.1-like | Indonesia | Q | Mar 2021 | 19 | 20 | 20 |
|  | 22 | Q6 | B.1 |  | Indonesia | Q | Mar 2021 |  | 1 | 2 |
|  | 22 | Q7 | B.1.1.7 | Alpha | Philippines | Q | Mar 2021 |  | 13 | 13 |
|  | 22 | Q8 | B.1.1.7 | Alpha | Philippines | Q | Mar 2021 |  | 13 | 13 |
|  | 22 | Q9 | B.1.1.7 | Alpha | Philippines | Q | Mar 2021 |  | 13 | 14 |
|  | 23 | R3 | B.1.36 |  | India | R | Sep 2020 |  | 10 | 11 |
|  | 23 | R4 | B.1.36 |  | India | R | Sep 2020 |  | 10 | 11 |
|  | 24 | R10 | B.1.1.7 | Alpha | India | R | Apr 2021 |  | 5 | 5 |
|  | 24 | R11 | B.1.617.1 | B.1.617.1-like | India | R | Apr 2021 | 6 | 7 | 7 |
|  | 24 | R12 | B.1.617.1 | B.1.617.1-like | India | R | Apr 2021 | 5 | 5 | 7 |
|  | 24 | R13 | B.1.617.1 | B.1.617.1-like | India | R | Apr 2021 |  | 6 | 8 |
|  | 24 | R14 | B.1.617.1 | B.1.617.1-like | India | R | Apr 2021 |  | 7 | 8 |
|  | 24 | R15 | B.1.617.1 | B.1.617.1-like | India | R | Apr 2021 |  | 6 | 8 |
|  | 24 | R16 | B.1.1.7 | Alpha | Philippines | R | Mar 2021 |  | 12 | 13 |
|  | 24 | R17 | B.1.617.1 | B.1.617.1-like | India | R | Apr 2021 |  | 11 | 11 |
|  | 24 | R18 | B.1.617.2 | Delta | India | R | Apr 2021 |  | 12 | 13 |
|  | 24 | R19 | B.1.617.1 | B.1.617.1-like | India | R | Apr 2021 |  | 13 | 14 |
|  | 24 | R20 | B.1.617.1 | B.1.617.1-like | India | R | Apr 2021 |  | 13 | 14 |
|  | 24 | R21 | B.1.617.1 | B.1.617.1-like | India | R | Apr 2021 |  | 13 | 14 |
|  | 24 | R22 | B.1.617.1 | B.1.617.1-like | India | R | Apr 2021 |  | 13 | 14 |
|  | 24 | R23 | B.1.617.1 | B.1.617.1-like | India | R | Apr 2021 |  | 13 | 14 |
|  | 24 | R24 | B.1.617.1 | B.1.617.1-like | India | R | Apr 2021 |  | 13 | 14 |
|  | 24 | R25 | B.1.617.1 | B.1.617.1-like | India | R | Apr 2021 |  | 13 | 14 |
|  | 24 | R26 | B.1.617.1 | B.1.617.1-like | India | R | Apr 2021 |  | 14 | 16 |
|  | 24 | R27 | B.1.617.2 | Delta | India | R | Apr 2021 |  | 12 | 13 |
|  | 24 | R7 | B.1.1.7 | Alpha | India | R | Mar 2021 |  | 12 | 13 |
|  | 24 | R8 | B.1.1.7 | Alpha | India | R | Mar 2021 |  | 12 | 13 |
|  | 24 | R9 | B.1.351 | Beta | Philippines | R | Mar 2021 |  | 12 | 14 |
|  | 25 | S1 | B.1 |  | USA | S | Oct 2020 | 9 | 10 | 11 |
|  | 25 | S2 | B.1 |  | Pakistan | S | Oct 2020 | 8 | 10 | 11 |
|  | 26 | S3 | B.1.1.7 | Alpha | United Kingdom | S | Dec 2020 | 3 | 3 | 5 |
|  | 26 | S4 | B.1.1.7 | Alpha | UK | S | Dec 2020 | 6 | 8 | 9 |
|  | 26 | S5 | B.1.1.7 | Alpha | United Kingdom | S | Dec 2020 |  | 8 | 9 |
|  | 26 | S6 | B.1.1.216 |  | India | S | Dec 2020 |  | 12 | 13 |
|  | 27 | S7 | B.1.1.12 |  | Indonesia | S | Jan 2021 | 3 | 14 | 13 |
|  | 27 | S8 | B.1.1.63 |  | Philippines | S | Jan 2021 |  | 12 | 13 |
|  | 28 | S10 | B.1.1.7 | Alpha | Philippines | S | Mar 2021 | 7 | 12 | 13 |
|  | 28 | S11 | B.1.1.7 | Alpha | Philippines | S | Mar 2021 |  | 13 | 14 |
|  | 28 | S12 | B.1.1.7 | Alpha | Philippines | S | Mar 2021 | 10 | 12 | 13 |
|  | 28 | S13 | B.1 |  | Indonesia | S | Mar 2021 |  | 12 | 13 |
|  | 28 | S14 | B.1.1.7 | Alpha | Philippines | S | Mar 2021 |  | 13 | 14 |
|  | 28 | S15 | B.1.1.7 | Alpha | India | S | Mar 2021 |  | 12 | 13 |
|  | 28 | S16 | B.1.351 | Beta | Philippines | S | Mar 2021 |  | 12 | 13 |
|  | 28 | S17 | B.1.617.1 | B.1.617.1-like | India | S | Apr 2021 | 4 | 5 | 6 |
|  | 28 | S18 | B.1.617.1 | B.1.617.1-like | India | S | Apr 2021 | 6 | 6 | 8 |
|  | 28 | S19 | B.1.1.7 | Alpha | Philippines | S | Apr 2021 |  | 12 | 13 |
|  | 28 | S20 | B.1.617.1 | B.1.617.1-like | India | S | Apr 2021 |  | 14 | 14 |
|  | 28 | S21 | B.1.617.1 | B.1.617.1-like | India | S | Apr 2021 |  | 13 | 14 |
|  | 28 | S22 | B.1.617.1 | B.1.617.1-like | India | S | Apr 2021 |  | 14 | 14 |
|  | 28 | S23 | B.1.617.1 | B.1.617.1-like | India | S | Apr 2021 |  | 13 | 14 |
|  | 28 | S24 | B.1.617.1 | B.1.617.1-like | India | S | Apr 2021 |  | 9 | 9 |
|  | 28 | S25 | B.1.617.2 | Delta | Nepal | S | Apr 2021 | 5 | 7 | 9 |
|  | 28 | S26 | B.1.617.2 | Delta | Nepal | S | Apr 2021 | 5 | 8 | 9 |
|  | 28 | S27 | B.1.466.2 |  | Indonesia | S | Apr 2021 |  | 13 | 14 |
|  | 28 | S9 | B.1.525 | Eta | Togo | S | Mar 2021 |  | 13 | 14 |
|  | 29 | W1 | B.1.466.2 |  | Indonesia | W | Mar 2021 |  | 12 | 13 |
|  | 29 | W2 | B.1.466.2 |  | Indonesia | W | Mar 2021 | 11 | 12 | 13 |
|  | 29 | W3 | B.1.1.7 | Alpha | Philippines | W | Apr 2021 | 11 | 13 | 13 |
|  | 29 | W4 | B.1.351 | Beta | Philippines | W | Apr 2021 |  | 13 | 14 |
|  | 30 | X1 | B.1.1.7 | Alpha | Philippines | X | Mar 2021 | 8 | 13 | 13 |
|  | 30 | X2 | B.1.351 | Beta | Philippines | X | Mar 2021 |  | 13 | 13 |
|  | 30 | X3 | B.1.1.7 | Alpha | Philippines | X | Mar 2021 |  | 12 | 13 |
|  | 30 | X4 | B.1.351 | Beta | Philippines | X | Mar 2021 | 7 | 12 | 13 |
|  | 30 | X5 | B.1.1.7 | Alpha | Philippines | X | Mar 2021 |  | 13 | 13 |
|  | 30 | X6 | B.1.617.1 | B.1.617.1-like | India | X | Apr 2021 |  | 21 | 22 |
|  | 31 | Y1 | B.1.1.25 |  | Bangladesh | Y | Nov 2020 |  | 2 | 3 |
|  | 31 | Y2 | B.1.177 |  | United Kingdom | Y | Nov 2020 |  | 12 | 13 |
|  | 31 | Y3 | B.1 |  | USA | Y | Nov 2020 |  | 12 | 13 |
|  | 32 | Z1 | B.1.36 |  | Nepal | Z | Jan 2021 |  | 14 | 14 |
|  | 32 | Z2 | B.1.468 |  | Nepal | Z | Dec 2020 | 18 | 20 | 20 |
|  | 32 | Z3 | B.1.468 |  | Indonesia | Z | Jan 2021 |  | 12 | 13 |
|  | 33 | A1 | B.1.1.7 | Alpha | Pakistan | A | Apr 2021 |  | 12 | 13 |
|  | 33 | A2 | B.1.466.2 |  | Indonesia | A | Apr 2021 | 3 | 13 | 14 |
|  | 34 | F1 | B.1.351 | Beta | Philippines | F | Mar 2021 | 9 | 12 | 13 |
|  | 34 | F2 | AY.75 | Delta | Nepal | F | Apr 2021 | 6 | 8 | 8 |
|  | 35 | L1 | B.1.617.1 | B.1.617.1-like | India | L | Apr 2021 |  | 10 | 10 |
|  | 35 | L2 | B.1.617.1 | B.1.617.1-like | India | L | Apr 2021 |  | 11 | 12 |
|  | 35 | L3 | B.1.617.1 | B.1.617.1-like | India | L | Apr 2021 |  | 11 | 12 |
|  | 35 | L4 | B.1.617.1 | B.1.617.1-like | India | L | Apr 2021 |  | 12 | 14 |
|  | 35 | L5 | B.1.1.7 | Alpha | Japan | L | Apr 2021 |  | 14 | 15 |
|  | 35 | L6 | B.1.617.2 | Delta | India | L | Apr 2021 |  | 12 | 13 |
|  | 36 | M12 | AY.71 | Delta | India | M | Apr 2021 | 6 | 13 | 13 |
|  | 36 | M13 | B.1.351 | Beta | Kenya | M | Apr 2021 |  | 13 | 14 |
|  | 36 | M14 | B.1.617.1 |  | India | M | Apr 2021 |  | 24 | 24 |
|  | 36 | M15 | B.1.351 | Beta | Philippines | M | Apr 2021 | 5 | 8 | 9 |
|  | 36 | M16 | AY.75 | Delta | Nepal | M | Apr 2021 | 7 | 7 | 8 |
|  | 36 | M17 | AY.75 | Delta | Nepal | M | Apr 2021 | 6 | 7 | 9 |
|  | 36 | M18 | AY.75 | Delta | Nepal | M | Apr 2021 |  | 8 | 9 |
|  | 36 | M19 | B.1.36.27 |  | Nepal | M | Apr 2021 |  | 8 | 9 |
|  | 36 | M20 | B.1.617.2 | Delta | Nepal | M | Apr 2021 |  | 8 | 9 |
|  | 36 | M21 | B.1.617.2 | Delta | Nepal | M | Apr 2021 | 4 | 7 | 9 |
|  | 36 | M22 | AY.75 | Delta | Nepal | M | Apr 2021 |  | 8 | 9 |
|  | 36 | M23 | AY.75 | Delta | Nepal | M | Apr 2021 | 4 | 8 | 9 |
|  | 36 | M24 | B.1.1.7 | Alpha | Nepal | M | Apr 2021 |  | 8 | 9 |
|  | 36 | M25 | B.1.1.7 | Alpha | Nepal | M | Apr 2021 | 3 | 8 | 9 |
|  | 36 | M26 | B.1.617.2 | Delta | Nepal | M | Apr 2021 |  | 7 | 8 |
|  | 37 | O1 | BA.1 | Probable Omicron | Ireland | O | Dec 2021 |  | 5 | 6 |
|  | 37 | O2 | BA.1 | Probable Omicron | United Kingdom | O | Dec 2021 | 2 | 2 | 3 |
|  | 37 | O3 | BA.1.1 | Probable Omicron | Australia | O | Dec 2021 |  | 2 | 3 |
|  | 37 | O4 | BA.1.1 | Probable Omicron | Kazakhstan | O | Dec 2021 |  | 3 | 4 |
|  | 37 | O5 | BA.1.17.2 | Probable Omicron | Pakistan | O | Dec 2021 | 4 | 4 | 6 |
|  | 37 | O6 | BA.1.1 | Omicron | USA | O | Dec 2021 |  | 2 | 3 |
|  | 37 | P1 | B.1.617.2 | Delta | Korea | P | Oct 2021 |  | 2 | 4 |
|  | 37 | P10 | B.1.617.2 | Delta | Finland | P | Nov 2021 | 5 | 7 | 8 |
|  | 37 | P11 | AY.33 | Delta | Tanzania | P | Dec 2021 | 2 | 2 | 3 |
|  | 37 | P2 | B.1.617.2 | Delta | Korea | P | Oct 2021 |  | 2 | 4 |
|  | 37 | P3 | AY.38 | Delta | Philippines | P | Oct 2021 |  | 3 | 4 |
|  | 37 | P4 | AY.99 | Delta | Ukraine | P | Oct 2021 |  | 2 | 4 |
|  | 37 | P5 | B.1.617.2 | Delta | Saudi Arabia | P | Oct 2021 | 6 | 7 | 9 |
|  | 37 | P6 | AY.69 | Delta | Korea | P | Nov 2021 | 9 | 10 | 11 |
|  | 37 | P7 | AY.127 | Delta | Singapore | P | Nov 2021 |  | 1 | 2 |
|  | 37 | P8 | AY.46.5 | Delta | Pakistan | P | Nov 2021 | 5 | 7 | 9 |
|  | 37 | P9 | AY.23 | Delta | Pakistan | P | Nov 2021 |  | 10 | 11 |
|  | 38 | Q11 | B.1.1.7 | Alpha | Turkey | Q | Apr 2021 | 3 | 9 | 10 |
|  | 38 | Q12 | B.1.617.2 | Delta | India | Q | Apr 2021 |  | 12 | 13 |
|  | 38 | Q13 | B.1.351 | Beta | Philippines | Q | Apr 2021 |  | 10 | 12 |
|  | 38 | Q14 | B.1.466.2 |  | Indonesia | Q | Apr 2021 |  | 5 | 6 |
|  | 39 | U1 | B.1.351 | Beta | Philippines | U | Mar 2021 | 9 | 12 | 13 |
|  | 39 | U2 | B.1.617.2 | Delta | India | U | Apr 2021 | 10 | 11 | 13 |
|  | 39 | U3 | AY.75 | Delta | India | U | Apr 2021 |  | 13 | 14 |
|  | 39 | U4 | B.1.470 |  | Indonesia | U | Apr 2021 | 10 | 12 | 17 |
|  | 40 | W5 | AY.108 | Delta | Pakistan | W | Oct 2021 |  | 3 | 4 |
|  | 40 | W6 | AY.108 | Delta | Pakistan | W | Oct 2021 |  | 3 | 6 |
|  | 41 | Y4 | B.1.617.2 | Delta | Philippines | Y | Sep 2021 |  | 3 | 4 |
|  | 41 | Y5 | B.1.617.2 | Delta | Philippines | Y | Sep 2021 |  | 3 | 4 |
|  | 41 | Y6 | B.1.617.2 | Delta | Philippines | Y | Sep 2021 |  | 3 | 4 |
|  | 42 | Z4 | B.1.1.7 | Alpha | Pakistan | Z | Mar 2021 |  | 4 | 5 |
|  | 42 | Z5 | B.1.1.7 | Alpha | Pakistan | Z | Mar 2021 |  | 13 | 13 |
|  | 42 | Z6 | B.1.1.7 | Alpha | Pakistan | Z | Mar 2021 |  | 13 | 13 |
|  | 42 | Z7 | B.1.351 | Beta | Pakistan | Z | Mar 2021 | 12 | 13 | 15 |
|  | 42 | Z8 | B.1.351.3 | Beta | Bangladesh | Z | Mar 2021 |  | 12 | 13 |
|  | 43 | G10 | AY.43 | Delta | United Kingdom | G | Dec 2021 | 2 | 3 | 4 |
|  | 43 | G11 | AY.43 | Delta | Czech | G | Nov 2021 |  | 9 | 10 |
|  | 43 | G12 | BA.2.3 | Omicron | Sweden | G | Jan 2022 |  | 3 | 4 |
|  | 44 | I1 | AY.43 | Delta | United Kingdom | I | Nov 2021 |  | 3 | 4 |
|  | 44 | I2 | AY.25 | Delta | USA | I | Nov 2021 |  | 4 | 4 |
|  | 44 | I3 | AY.4.2 | Delta | United Kingdom | I | Nov 2021 | 3 | 5 | 6 |
|  | 45 | I4 | BA.2.12 | Omicron | India | I | Jan 2022 | 2 | 4 | 4 |
|  | 45 | I5 | BA.1 | Omicron | Italy | I | Jan 2022 | 4 | 9 | 10 |
|  | 46 | K1 | BA.1.17.2 | Omicron | Canada | K | Dec 2021 | 3 | 4 | 5 |
|  | 46 | K2 | BA.2 | Omicron | Australia | K | Jan 2022 | 2 | 3 | 4 |
|  | 46 | K3 | BA.1.17 | Omicron | Switzerland | K | Jan 2022 |  | 5 | 6 |
|  | 47 | N1 | BA.1.21 | Omicron | Ghana | N | Dec 2021 |  | 3 | 4 |
|  | 47 | N2 | BA.1.1 | Probable Omicron | Morocco | N | Dec 2021 |  | 5 | 6 |
|  | 48 | V1 | BA.1.17.2 | Omicron | Philippines | V | Jan 2022 |  | 4 | 4 |
|  | 48 | V2 | BA.2.3 | Omicron | Philippines | V | Jan 2022 | 2 | 4 | 4 |
|  | 49 | X7 | BA.1 | Omicron | Nepal | X | Jan 2022 |  | 6 | 6 |
|  | 49 | X8 | BA.2.2 | Omicron | Nepal | X | Jan 2022 | 4 | 5 | 7 |
